# Pyroxylin shortens the resting stage of the hair cycle in mice

**DOI:** 10.1038/s41598-026-52804-0

**Published:** 2026-05-11

**Authors:** Shoichiro Kokabu, Kunikazu Tsuji, Chou Ying-Ying, Ayako Washio, Tomohiko Shirakawa, Yusuke Ono, Quan Yuan, Osamu Kaminuma, Takuma Matsubara

**Affiliations:** 1 Division of Biochemistry, Kyushu Dental University, Kitakyushu, Fukuoka, 803-8580 Japan; 2 Department of Orthopedic Surgery, Tokyo Medical and Dental University, Bunkyo, Tokyo, 113-8519 Japan; 3 Division of Endodontics and Restorative Dentistry, Kyushu Dental University, Kitakyushu, Fukuoka, 803-8580 Japan; 4 Division of Orofacial Functions and Orthodontics, Faculty of Dentistry, Kyushu Dental University, Kitakyushu, Fukuoka, 803-8580 Japan; 5 Department of Muscle Development and Regeneration, Institute of Molecular Embryology and Genetics (IMEG), Kumamoto University, Kumamoto, 860-0811 Japan; 6 Tokyo Metropolitan Institute for Geriatrics and Gerontology, Itabashi, Tokyo, 173-0015 Japan; 7 State Key Laboratory of Oral Diseases & National Center for Stomatology & National Clinical Research Center for Oral Diseases, West China Hospital of Stomatology, Sichuan University, Chengdu, China; 8 Department of Disease Model, Research Institute of Radiation Biology and Medicine, Hiroshima University, Hiroshima, 734-8551 Japan; 9 Kyushu Dental University Oral Medicine Innovation Center, Kitakyushu, Fukuoka, 803-8580 Japan

**Keywords:** Biological techniques, Biotechnology

## Abstract

Despite strong interest in scalp and body hair and a large hair-care market, effective and practical approaches for inducing hair growth remain limited. Here, we show that pyroxylin treatment shortens the resting stage of the hair cycle and induces localized hair growth in mice. Application of pyroxylin to various skin regions induced injury followed by hair growth, which was also triggered by other adhesive materials. The hair growth was accompanied by hair cycle-related gene expression and gradually synchronized with the cycle of surrounding hair growth. Notably, this approach was effective even in middle-aged and aged mice. Although direct comparisons with established wound-healing models and physiological hair-cycle systems will be needed to further define the scope and limitations of this approach, our findings suggest that pyroxylin treatment provides a useful experimental platform for studying localized hair growth, and they warrant further investigation of its potential for topical hair-growth induction.

**Supplementary Information:**

The online version contains supplementary material available at 10.1038/s41598-026-52804-0.

## Introduction

Interest in scalp and body hair is high among both women and men. Globally, the market is valued at $7.6 billion and is projected to reach $13 billion by 2028^1^. Nevertheless, despite intense research and development competition, a highly effective, practical hair-regeneration therapy has yet to be developed^2^. Hair follicles formed during embryogenesis undergo lifelong cycles of growth (anagen), regression (catagen), and rest (telogen)—the hair cycle^3^. In common presentations of hair thinning and alopecia, hairs enter regression before achieving sufficient growth during anagen, the resting phase is prolonged, and follicles undergo miniaturization with resulting hair loss^4,5^. Thus, developing new treatments for thinning and alopecia requires experimental models that accurately capture the transitions from the regenerative phase, through regression, to the resting phase.

Although mice are widely used to study disease pathogenesis and for drug discovery, they are often considered unsuitable for hair research. In mice, anagen lasts 17–21 days and catagen approximately 3–4 days, whereas telogen is very long^6–8^. In commonly used C57BL/6 and C3H/He female mice, the second telogen lasts 7 and 14 weeks, respectively. Consequently, due to the prolonged duration of telogen, it is challenging to determine the precise timing of the transition from telogen to anagen. Furthermore, although hair follicles in the dorsal skin are largely synchronized during the first postnatal month, the hair cycle becomes increasingly complex across strains and body sites^9^. This complexity is thought to reflect locally reactive hair growth, which occurs independent of the body-wide cycle, as well as locally coordinated hair cycles^10^.

Methods to trigger hair growth from telogen follicles at a specific time include plucking-induced hair growth (PIHG) and wound-induced hair growth (WIHG). In both paradigms, local inflammatory stimuli recruit macrophages and, via inflammatory cytokines and activation of Wnt signaling, activate hair follicle stem cells (HFSCs) to enter anagen^11^. In PIHG, hairs are reliably removed using wax. However, this procedure is labor- and time-intensive, and it is difficult to quantitatively assess both the extent and density of the plucked area. Previous studies on WIHG have shown that skin injury can influence the behavior of surrounding hair follicles, including the activation of resting follicles and the induction of regenerative, hair growth-associated responses. These observations support the concept that injury-associated signals can modulate local hair cycle progression and hair follicle stem cell activity in adjacent skin^12–14^.

Tape-stripping assays were originally developed as a reproducible model of superficial epidermal injury and barrier disruption and have been widely used to investigate skin barrier function and wound healing while preserving the underlying dermis^15,16^. More recently, tape-stripping–based epidermal abrasion has been shown to activate and mobilize HFSCs during barrier repair^17^ and, under certain conditions, to induce TRPV1-dependent hair growth in mice^18^.

However, the irregular shape of the induced hair-growth by these approaches makes quantitative evaluation difficult. In addition, because this method involves creating a skin defect—an invasive manipulation—it may raise concerns about potential systemic effects and an increased risk of infection. Moreover, few studies have tracked anagen induced by these approaches through catagen and into the subsequent telogen^19^.

In the independent biocompatibility experiment involving subcutaneous implantation of biomaterials, we closed dorsal skin incision in mice using a pyroxylin-based tissue adhesive instead of sutures. Surprisingly, pronounced and sharply demarcated hair regrowth was observed only in the adhesive-coated area, while the adjacent shaved skin showed no regrowth during the same period.

In this study, we describe a simple mouse model in which topical application of pyroxylin and related adhesive materials shortens the resting stage of the hair cycle. This approach allows observation of the telogen-to-anagen transition at defined time points and facilitates post-treatment analyses of hair cycle dynamics.

## Results

### Pyroxylin and related adhesive materials induce localized hair growth

**Fig. 1:**
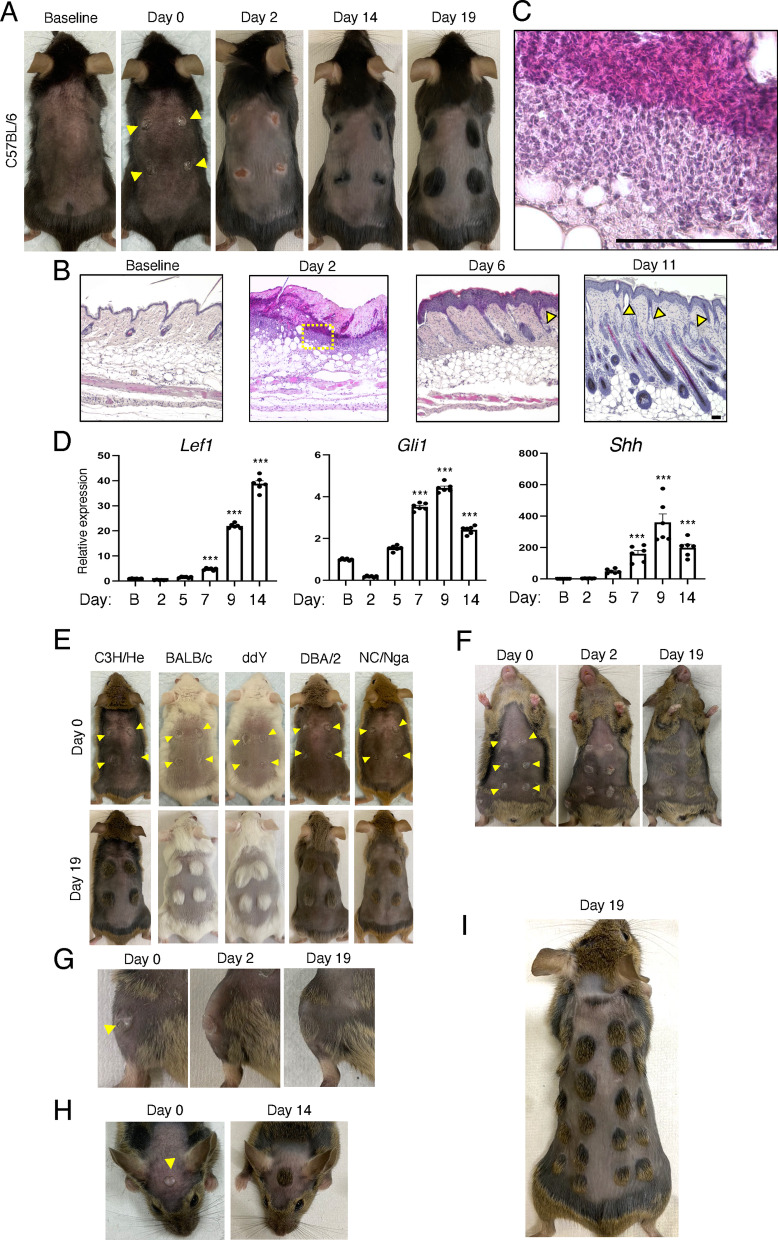
Pyroxylin induces skin wounds followed by hair growth. (**A**) Ten percent pyroxylin solution was applied to the dorsal skin of 8-week-old female C57BL/6N mice after shaving (yellow arrowhead). Representative dorsal skin images of 8-week-old female mice at baseline (before application) and at day 0 (immediately after pyroxylin application), day 2, day 14, and day 19. (**B**) Representative H&E staining images of dorsal back skin wounds at baseline, and at days 1, 2, 6, and 11 are shown. Scale bars indicate 100 μm. Yellow dotted lines delineate areas of inflammatory cell infiltrates in the upper dermis, and arrowheads indicate sebaceous glands. (**C**) Higher-magnification view of the area outlined by the yellow dashed line in the day 2 image in (**B**). The scale bar is the same as in (**B**). (**D**) mRNA levels of Lef1, Gli1, and Shh were determined by real-time PCR from dorsal skin wounds collected at baseline (B), and at day 1, 2, 5, 7, 9, and 14. (**E**) Pyroxylin was applied to the dorsal skin of 8-week-old female C3H/He, BALB/c, ddY, DBA/2, and NC/Nga mice after shaving (yellow arrowhead). Representative photos on days 0, 2, 14, and 19 are shown. (**F**–**I**) Pyroxylin was applied to the skin of the abdomen (**F**), lower leg (**G**), and head (**H**) after shaving (yellow arrowhead), and at 20 points on the dorsal skin (I). Representative photos on days 0, 2, 14, and/or 19 are shown. (**A**–**C**, **E**–**I**) Representative images from 6 individual mice are shown. (**D**) Mean ± SEM; *n* = 6. *** *p* < 0.001, compared with baseline.

To determine whether topical application of pyroxylin induces localized hair growth in telogen-phase dorsal skin, we shaved the dorsal skin of 8-week-old female mice and applied pyroxylin to four predefined circular areas. At this age and anatomical site, dorsal hair follicles are predominantly in telogen, and shaving alone does not induce appreciable hair regrowth for at least 3–4 weeks. Consistent with this, shaved but untreated skin showed no visible hair regrowth during the observation period. In contrast, pigmented hair progressively appeared within the pyroxylin-treated areas, whereas the surrounding shaved skin remained largely hairless. By day 19, hair length and density in the treated regions had nearly recovered to levels comparable to those of the unshaved coat. These findings indicate that topical application of pyroxylin induces robust and spatially restricted hair growth in telogen dorsal skin (Fig. 1A).

We next performed histological analysis during pyroxylin-induced wound generation and hair regeneration in the dorsal skin. On day 2, marked inflammatory cell accumulation was observed in the epidermis and underlying dermis (Fig. 1B and C, Supplementary Fig. 1). In addition to epidermal regeneration, the growth of hair follicles accompanied by sebaceous glands, indicated by yellow arrowheads, was observed on days 6 and 11. Hair follicles elongated toward the subcutaneous adipose tissue, with hair shafts growing thicker and longer on day 11 (Fig. 1B). mRNA expression analysis with quantitative real-time PCR revealed that expression of *Lef1* (Wnt), *Gli1* (Hedgehog), and *Shh* (Sonic hedgehog) increased over time as hair regeneration progressed (Fig. 1D).

The hair cycle, including the length and timing of telogen and anagen, varies between the sexes and in mouse strains. In addition to C57BL/6, C3H/He has been used for wound healing and hair growth research^20,21^. The hair cycles of these two mouse strains are different. However, there was no significant difference in the level or time course of hair regeneration following pyroxylin application between the C57BL/6N and C3H/He mouse strains (Fig. 1A and E) or between male and female mice (data not shown).

The abdominal and limb hair characteristics significantly differ from those of the dorsal hair. The skin of the head and back develops from distinct germ layers during embryonic development^22,23^. Therefore, pyroxylin-induced hair growth was examined in these regions of C3H/He mice, which are characterized by a long telogen period and a tightly synchronized hair cycle. Following shaving, pyroxylin application to the abdomen (Fig. 1F), lower limb (Fig. 1G), and head (Fig. 1H) induced hair growth in all regions with a time course similar to that observed in the dorsal skin. However, in addition to the lack of beard regeneration, hair regeneration was not observed in hairless regions, such as the plantar area, following pyroxylin application (data not shown). Using this method, hair growth could be evaluated at least 20 sites on the dorsal skin of a single mouse (Fig. 1I).

**Fig. 2:**
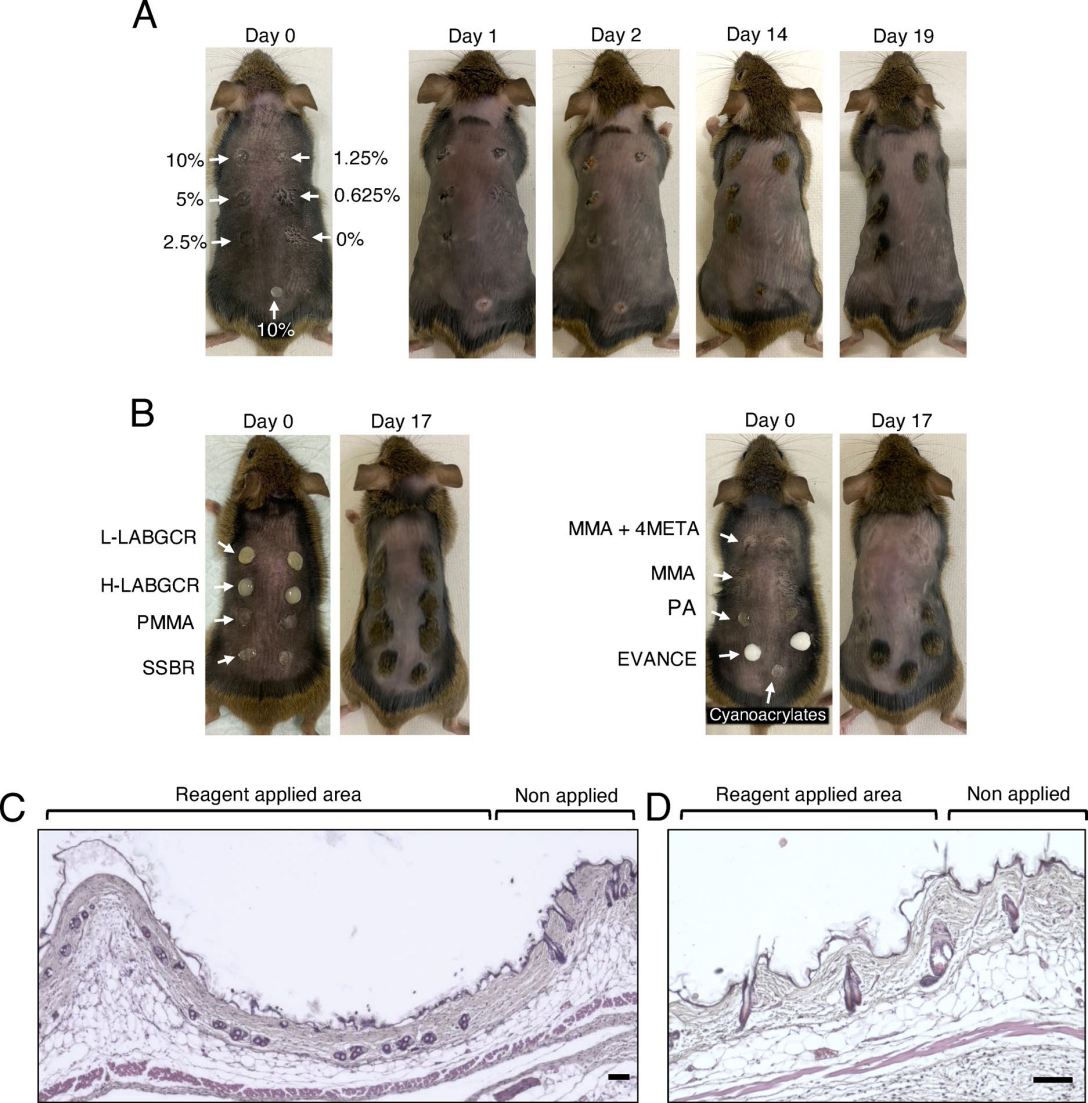
Adhesive materials induce hair growth. (**A**) Zero (ethanol:diethyl ether = 1:1 weight), 0.625, 1.25, 2.5, 5, or 10% pyroxylin solution was applied (yellow arrowhead) to the dorsal skin of 8-week-old C3H/He mice after shaving. Representative photos on days 0, 2, 14, and 19 are shown. (**B**) After shaving, low- or high-viscosity light-activated Bis-GMA-based composite resins (L-LABGCR or H-LABGCR), PMMA, SSBR, MMA, MMA + 4-META, PA, EVACE, and cyanoacrylates were applied to the dorsal skin. Representative photos of three independent experiments are shown. (**C** and **D**) Representative H&E staining images of wounds on dorsal back skin 3 h after pyroxylin application (**C**) and a corresponding magnified image (**D**) are shown. Representative images with the same trend from 6 individual mice are shown. Scale bars indicate 100 μm.

The dose-dependent ability of pyroxylin to induce hair growth was examined. Pyroxylin at concentrations greater than 1.25% induced essentially the same level of hair growth, although obvious wound and hair regeneration were not observed at concentrations less than 0.625% (Fig. 2A). The effects of adhesive materials other than pyroxylin were examined. The application of bisphenol A-glycidyl methacrylate (Bis-GMA)-based composite resins with low- or high-viscosity, polymethyl methacrylate (PMMA), solvent-based styrene-butadiene rubber (SSBR), and cyanoacrylates created wounds and induced hair growth in the dorsal skin, similar to those observed with pyroxylin treatment. Water-soluble materials such as ethylene–vinyl acetate copolymer emulsions (EVACE) and polyvinyl alcohol aqueous solutions (PA) can also induce hair growth. However, application of pure liquid components, including methyl methacrylate (MMA) + 4-methacryloxy ethyl trimelitate anhydride (4-META) or MMA alone, did not induce hair growth (Fig. 2B). Three hours after pyroxylin treatment, prominent epidermal alterations were observed predominantly within the treated area (Fig. 2C, D). These findings suggest that pyroxylin treatment promotes the transition from telogen to anagen at the treated site. Accordingly, this approach provides a useful model for analyzing localized hair growth and hair cycle progression after treatment.

### Hair growth after pyroxylin application was also observed in middle-aged and aged mice

**Fig. 3:**
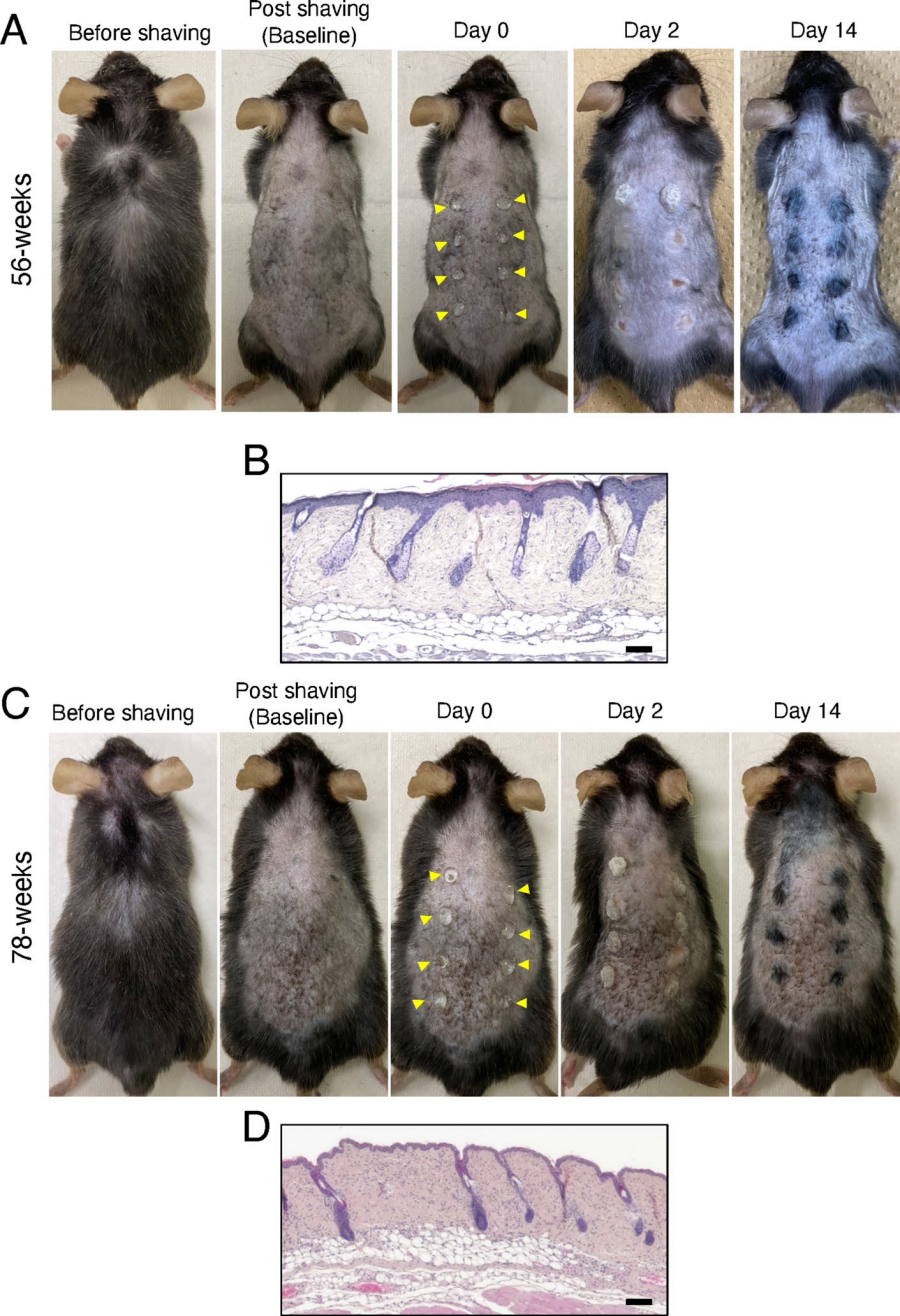
Hair growth after pyroxylin application in middle-aged and aged mice. (**A**–**D**) Ten percent pyroxylin solution was applied (yellow arrowhead) to the dorsal hair of middle-aged (56-weeks) (**A** and **B**) and aged (78-weeks) (**C** and **D**) male C57BL/6J mice before or after shaving. (**A** and **C**) Representative photos on days 0, 2, and 14 are shown. (**B** and **D**) Representative H&E staining images of dorsal back skin wounds on day 6 are shown. Scale bars indicate 100 μm. (**A**–**D**) All mice examined exhibited the same trend (n = 6).

The demand for hair regenerative medicine is increasing in the middle-aged and aged life stages because hair loss is an age-related process^24^. Based on the correlation between mouse and human life spans, 56- and 78-week-old C57BL/6 J mice are regarded as middle-aged and aged, respectively^25^. The dorsal hair cycles of 56- and 78-week-old C57BL/6 J mice are in the 4th telogen phase^21^. We next examined whether pyroxylin application also induced hair growth in these mice. Pyroxylin-induced hair growth was observed in 56-week-old mice over the same time course as that observed in 8-week-old mice (Fig. 1A, B, 3A, B). Similar hair growth was also observed in 78-week-old mice after pyroxylin application (Fig. 3C, D).

### Pyroxylin treatment is associated with synchronization of the surrounding hair cycle

**Fig. 4:**
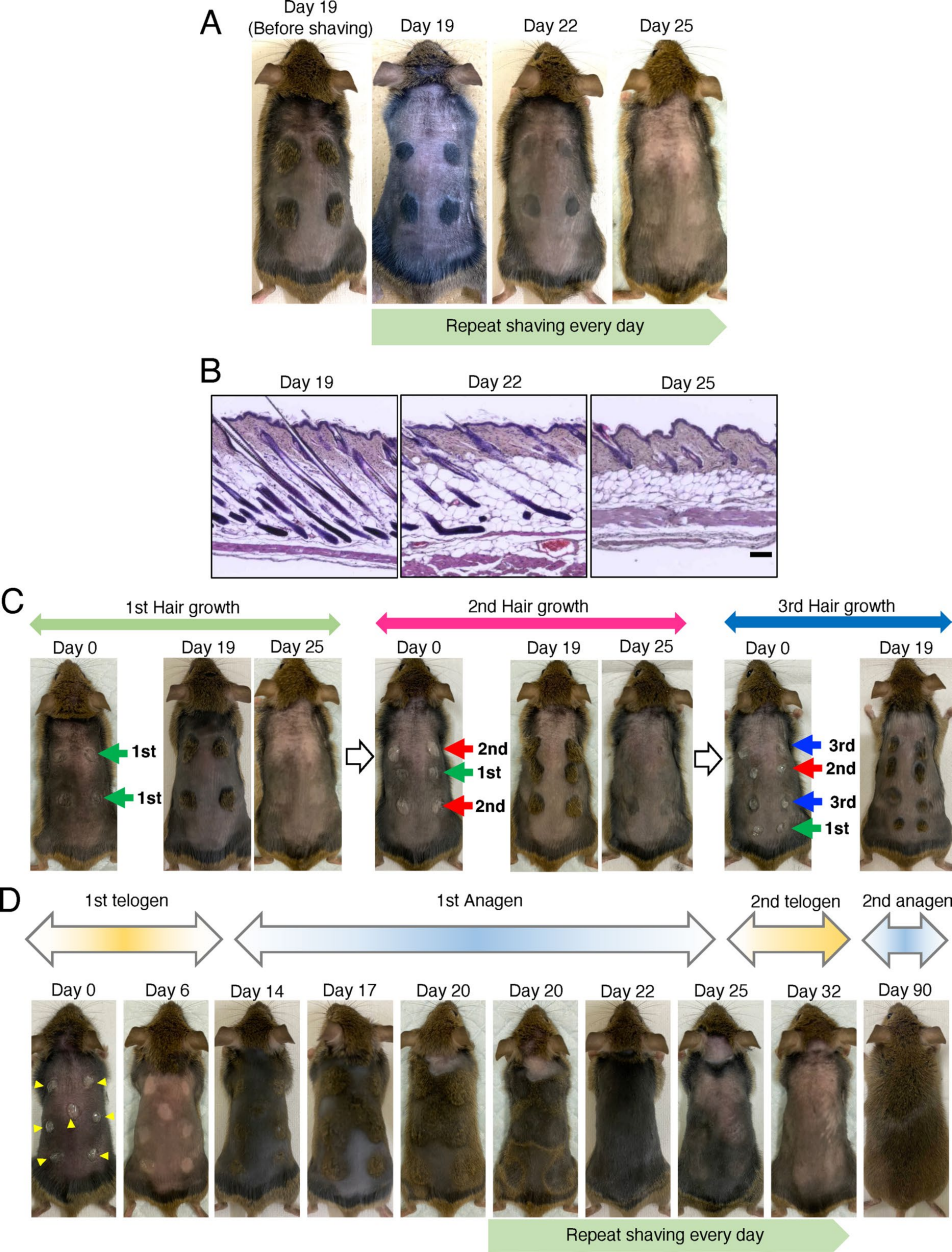
Hair changes in pyroxylin-treated and surrounding areas. (**A**) Ten percent pyroxylin solution was applied to the dorsal skin of 8-week-old female C3H/He mice after shaving. Beginning on day 19, the regenerated hairs were shaved every day. Representative photos on days 19, 22, and 25 are shown. (**B**) Representative H&E staining images of dorsal back skin wounds on days 19, 22, and 25 are shown. Scale bars, 100 µm. (**C**) Ten percent pyroxylin solution was applied to the dorsal skin of 8-week-old female C3H/He mice after shaving (green arrow). The photos are shown on day 0, 19, or 25 (1st Hair growth). A 10% pyroxylin solution was also applied at the same location for the first time (2nd time; red arrow). The photos are shown on days 0, 19, and 25 (2nd Hair growth). Again, a 10% pyroxylin solution was applied to the same place for the 2nd time (3rd time; blue arrow). The photos are shown on days 0, 19, and 25 (3rd Hair growth). (**D**) Pyroxylin was applied to the dorsal hair of 4-week-old female C3H/He mice after shaving (yellow arrowhead). Beginning on day 20, the regenerated hair was shaved every day for 2 weeks. Representative photos on days 0, 6, 14, 17, 20, 22, 25, 32, and 90 are shown. All mice examined exhibited the same trend (n = 6).

We further examined subsequent hair growth following repeated shaving. After the regenerated hair in pyroxylin-treated regions had recovered to a length and density comparable to those of the unshaved coat, the hair was shaved daily. This process enabled the determination of the hair cycle based on the skin color. The black color of the hair regeneration area after shaving on day 19 gradually faded with each subsequent shaving. By the 25th day, the color became almost indistinguishable from the surrounding skin color (Fig. 4A). Consistent with the changes in skin color, the histological appearance of the pyroxylin-treated area changed markedly on days 19, 22, and 25 (Fig. 4B). At day 19, induced follicles remained in an active anagen stage, characterized by densely packed, elongated follicles with large hair bulbs extending deep into the subcutis. By day 22, many follicles displayed clear regressing catagen features, including shrinkage of the hair bulbs and upward retraction of the follicle base. By day 25, most follicles exhibited telogen morphology, appearing shortened and positioned more superficially, with compact bulbs located in the upper dermis. These observations indicate that the effects of pyroxylin are largely confined to the treated region, with follicles within and immediately adjacent to the treated area progressing from a growth phase through regression and returning to a telogen state.

The hair growth after repeated pyroxylin treatment was examined. During telogen, pyroxylin was reapplied to an area in which pyroxylin-induced hair growth had previously been observed. Hair growth following the second application was comparable to that observed after the first application, and a similar response was also observed after the third pyroxylin application (Fig. 4C). When pyroxylin was applied to 4-week-old C3H/He mice during the first physiological telogen phase, hair growth was observed approximately two weeks before the onset of the first physiological anagen phase. Following the onset of the first physiological anagen phase at approximately day 17, hair also began to grow in the shaved area without pyroxylin application. Based on skin color changes observed after daily shaving between days 20 and 32, the pyroxylin-treated area appeared to return to a resting state earlier than the surrounding untreated skin. However, this difference had largely disappeared by approximately day 90. Thus, by the second anagen phase, hair cycling in the pyroxylin-treated area became similar to that in the surrounding untreated skin (Fig. 4D).

## Discussion

In this study, we describe a simple and reproducible mouse model in which localized hair regeneration is induced by the topical application of pyroxylin and related adhesive materials to telogen-phase dorsal skin. Pyroxylin treatment consistently triggered a hair cycle within the treated area, as demonstrated by synchronized macroscopic hair regrowth, characteristic histological transitions from anagen to catagen and early telogen follicle states, and transient upregulation of hair cycle–related genes, including *Lef1*, *Gli1* and *Shh*. Notably, these responses were observed across multiple mouse strains and age groups, indicating that pyroxylin treatment provides a robust and broadly applicable platform for analyzing injury-induced hair growth under diverse genetic and physiological conditions.

Although the primary aim of the present study is not to propose pyroxylin treatment itself as an immediate therapy for human alopecia, the potential translational relevance of our findings warrants consideration. Pyroxylin has long been used clinically in the form of collodion or flexible collodion for wound covering and dressing fixation^26,27^, and pyroxylin-based liquid bandages have also been reported to promote skin wound healing^28^. In this context, our findings raise the possibility that pyroxylin-based topical formulations, or related film-forming materials with improved safety profiles and greater controllability, could eventually inform new localized strategies for hair growth induction. At the same time, substantial further studies will be required to establish efficacy and safety in human skin and clarify the depth of tissue effects, reproducibility, and overall translational feasibility.

Compared with PIHG, WIHG, and tape-stripping assays, this pyroxylin-based approach is technically straightforward, requiring only topical application of pyroxylin. In addition, the extent of the induced hair growth area can be modulated by adjusting the application area. Pyroxylin-induced hair growth aligns with superficial injury/barrier-disruption paradigms and may be regarded not as an entirely separate category, but as a variant within the spectrum of tape-stripping–type assays.

Macroscopic inspection and histological analysis indicated that, although the hair-growing area was sharply demarcated, it did not always coincide precisely with the geometric boundaries of pyroxylin application. In many cases, the hair-growing area extended slightly beyond the visibly treated region, forming a narrow “halo” in which follicles also showed anagen morphology. This pattern suggests that the effects of pyroxylin are not limited to follicles directly beneath the treated area but may extend a short distance to neighboring follicles and surrounding tissue components. Such behavior is reminiscent of the concept of hair follicle “quorum sensing,” in which the collective state of a local follicular community influences the initiation and spread of an anagen wave^11^. Although the present observations are primarily morphological, the frequent extension of the hair-growing area beyond the physical application boundary raises the possibility that local interactions among follicles and adjacent tissues contribute to defining the extent and boundaries of pyroxylin-induced hair growth.

In our model, once the treated area had returned to telogen, reapplication of pyroxylin reliably re-induced hair growth again, recapitulating the response observed after the initial treatment. As long as the skin remained in telogen, hair growth could be repeatedly induced (Fig. 4). Furthermore, our approach currently allows evaluation of up to 20 hair-growth sites on the dorsal skin of a single mouse (Fig. 1I). This capability markedly shortens the time required to observe hair growth and related changes and, by enabling the screening of multiple candidate treatments in a single animal, can substantially reduce the number of mice used and the overall observation period. In recent years, there has been a worldwide call to minimize animal use in experiments and to consider alternative methods^29,30^. However, compared with organ culture and cell-based systems, in vivo evaluation systems that faithfully reflect the complex biological responses involving cells and molecules remain, at present, indispensable^31^. Our model is not only technically simple but also aligns with ongoing efforts to reduce animal use, as it enables multiple analyses to be performed in a single animal and thereby reduces the total number of animals required.

Phenomena such as hair thinning, alopecia, and graying increase with age, concentrating the target population for hair-regeneration therapies in the mid- to late-life stages^32–34^. Accordingly, it is important to establish screening methods for identifying novel hair-growth–promoting approaches and agents for these populations. However, in contrast to young mice, which exhibit a relatively stable hair cycle, successful observation of hair regeneration and the hair cycle in middle-aged and older mice has been particularly limited^35–38^. In the present study, hair growth could be induced at defined time points in mice aged 56 and 78 weeks (Fig. 3). Experimental systems using HFSCs and organoids have been developed, but models that faithfully recapitulate aging remain inadequate^39,40^. In this context, the advances presented here are expected to deepen understanding of hair growth in aged environments and substantially accelerate studies using HFSCs and organoids that explicitly incorporate aging. Thus, this work is likely to contribute to the future development of hair-growth–promoting and hair-nourishing agents with reduced reliance on animal experimentation.

This study has several limitations. First, it provides a preliminary and primarily qualitative description of pyroxylin-induced hair growth rather than a comprehensive comparison with existing wound-healing and hair-growth models. We did not perform direct, side-by-side comparisons with established wound-healing, hair-regeneration, or physiological hair-cycle models. Accordingly, more detailed morphometric, molecular, and immunological analyses will be required to better characterize pyroxylin-induced hair growth and to define its relationship to models such as tape-stripping or excisional wounding. Second, the current analyses rely primarily on macroscopic observations, conventional histology, and bulk gene expression profiling. Higher-resolution approaches, including immunohistochemistry and single-cell or spatially resolved analyses, will be needed to better characterize local tissue changes, inflammatory responses, and cellular heterogeneity within and around the hair-growing area. Finally, we have not yet evaluated the effects of established hair-growth–promoting agents in this system. Therefore, the utility of this approach as a platform for pharmacological or genetic screening remains to be determined in future studies.

## Materials and methods

### Animals

Female mice were purchased from Japan SLC, Inc. (Hamamatsu, Japan). The mouse strains used were BALB/cCrSlc (RRID: MGI:2161019), Slc:ddY (RRID: MGI:5558113), DBA/2CrSlc (RRID: MGI:2160986), NC/NgaSlc (RRID: MGI:6323254), C3H/HeSlc (RRID: MGI:2165359) and C57BL/6NCrSlc (RRID: MGI:5295404). We also purchased 56- or 78-week-old male C57BL/6J (RRID: MGI:3028467) mice from The Jackson Laboratory Japan, Inc. (Yokohama, Japan). Body weight was recorded at the start of each experiment (prior to any procedure). Baseline body weight ranges (n = 6 per strain/age group) were as follows: BALB/cCrSlc (female), 17.7–19.3 g; Slc:ddY (female), 27.7–29.7 g; DBA/2CrSlc (female), 17.8–18.4 g; NC/NgaSlc (female), 20.6–25.4 g; C3H/HeSlc (8 weeks) (female), 19.0–21.3 g; C3H/HeSlc (P19) (female), 8.6–10.4 g; C57BL/6NCrSlc (8 weeks) (female), 17.5–20.4 g; C57BL/6J (56 weeks) (male), 31.1–34.6 g; and C57BL/6J (78 weeks) (male), 32.6–38.2 g. All animal experiments were approved by the Experimental Animal Care and Use Committee of Kyushu Dental University (approval numbers 22–004, 22–020, 22–022, 23–006, 23–014, and 23–016) and were conducted in accordance with the relevant guidelines and regulations. All animal studies also complied with the ARRIVE guidelines.

### Types and sources of adhesive materials

We evaluated the adhesive materials listed below as candidate materials for inducing hair growth. Selection criteria included the ability to form a continuous film on the skin surface after application, visible changes during drying, and the absence of components associated with severe systemic toxicity in mice. Various concentrations of pyroxylin solution (FUJIFILM Wako Chemicals, Osaka, Japan), hydrosoluble ethylene–vinyl acetate copolymer emulsion (EVACE) (ALTECO, Shiga, Japan), polyvinyl alcohol aqueous solution (PA) (Yamato Co., Ltd., Tokyo), cyanoacrylates (Toagosei Company, Limited, Tokyo, Japan), solvent-based styrene butadiene rubber (SSBR) (Cemedine Co., Ltd., Tokyo), light-activated bis-GMA-based composite resin (LABGCR) (Nippon Shika Yakuhin Co., Ltd., Shimonoseki), methyl methacrylate (MMA) (Shofu, Inc., Kyoto, Japan), 4-methacryloxy ethyl trimelitate anhydride (4-META) (Sun Medical Company), and polymethyl methacrylate (PMMA) powder (Sun Medical Company, Ltd., Shiga, Japan) mixed with MMA + 4META were used for dorsal application. PMMA powder was mixed with MMA and 4-META to initiate polymerization immediately before dorsal application. Unless otherwise specified, all adhesive materials were applied undiluted, as supplied by the manufacturers. The pyroxylin solution, which contained 9.5–10.5% (w/w) pyroxylin in an ethanol–diethyl ether mixture, was applied undiluted to the shaved dorsal skin as a thin, continuous layer covering a defined area (approximately 8 × 8 mm).

### Application of adhesive materials and sampling schedule

Mice were anesthetized under general anesthesia using a triad of anesthetics: medetomidine (Nippon Zenyaku Kogyo Co., Ltd., Fukushima, Japan) (0.75 mg/kg), midazolam (Astellas Pharma, Inc., Tokyo, Japan) (4 mg/kg), and butorphanol (Meiji Seika Pharma Co., Pharma, Inc., Tokyo, Japan) (5 mg/kg)^41^. The dorsal hair was shaved using clippers, and materials were applied as approximately 8-mm-diameter circular sites. Light-activated bis-GMA-based composite resins were polymerized by light-emitting diode irradiation immediately following application. In all experiments, “baseline” was defined as the time point immediately after shaving and before application of any adhesive materials, and “day 0” was defined as the time point immediately after application of pyroxylin or the other adhesive materials. Macroscopic skin images of dorsal skin were obtained under general anesthesia at baseline (before application) and at days 0, 2, 6, 14, 17, 19, 20, 22, 25, 32, and 90 after application. For histological analysis, skin samples from the treated dorsal area were collected at baseline, 3 h, and at days 2, 11, 19, 22, and 25 after application. For quantitative real-time PCR, dorsal skin samples were collected at baseline and at days 2, 5, 7, 9, and 14 after application.

### Histopathological examination

Mice were euthanized by carbon dioxide (CO_2_) inhalation in a cylindrical chamber (22 cm in diameter and 18 cm in height; approximately 6.8 L). CO_2_ was delivered at 3–4 L/min (approximately 44–59% of the chamber volume per minute) using a calibrated flow meter prior to tissue collection. Skin samples were collected and fixed with 4% paraformaldehyde (Nacalai Tesque, Inc., Kyoto, Japan) in PBS, dehydrated through an ethanol and xylene series, embedded in paraffin, and cut into 4-μm sections^42^. After deparaffinization, the sections were stained with hematoxylin (FUJIFILM Wako Chemicals) and eosin (FUJIFILM Wako Chemicals) (H&E). The sections were imaged with a Keyence BZ-X800 (Keyence, Osaka, Japan). The hair shafts were observed using a stereomicroscope (Leica EZ4 HD, Leica, Wetzlar, Germany).

### Quantitative real-time PCR

Total RNA was isolated from the skin tissues using a FastGeneTM RNA Basic Kit (Nippon Genetics, Tokyo, Japan) and reverse-transcribed into cDNA using a high-capacity cDNA reverse transcription kit (Applied Biosystems, Waltham, MA, USA). SYBR Green-based quantitative polymerase chain reaction (qPCR) was performed using PowerUp SYBR (Thermo Fisher Scientific, Waltham, MA, USA) and the QuantStudio 3 Real-Time PCR System (Thermo Fisher Scientific, Waltham, MA, USA). Relative quantification was performed by the ΔCT method using Tbp as the housekeeping gene for normalization. The primer sequences used for quantitative real-time PCR were as follows (5′–3′): *Lef1*, forward 5′-ACTGTCAGGCGACACTTCCATG-3′ and reverse 5′-GTGCTCCTGTTTGACCTGAGGT-3′; *Gli1*, forward 5′-CTCAAACTGCCCAGCTTAACCC-3′ and reverse 5′-TGCGGCTGACTGTGTAAGCAGA-3′; *Shh*, forward 5′-GGATGAGGAAAACACGGGAGCA-3′ and reverse 5′-TCATCCCAGCCCTCGGTCACT-3′; and *Tbp*, forward 5′-GGCGGTTTGGCTAGGTTT-3′ and reverse 5′-GGGTTATCTTCACACACCATGA-3′.

### Statistical analysis

qPCR data are expressed as the mean ± standard error of the mean (SEM) for the fold changes in gene expression compared with baseline. The data were analyzed using one-way ANOVA followed by Dunnett’s multiple comparisons test. *p* < 0.001 was considered to indicate statistical significance.

## Supplementary Information

Below is the link to the electronic supplementary material.

Supplementary Material 1

## Data Availability

The additional information required to reanalyze the data reported in this paper is available from the lead contact upon request.
