# Supplementary material for: Pyroxylin shortens the resting stage of the hair cycle in mice
Source: Sci Rep. 2026 May 11;16:21481. doi: 10.1038/s41598-026-52804-0 (PMC13350725; doi:10.1038/s41598-026-52804-0)
Supplement: Supplementary file 1 — Supplementary Material 1 [file 41598_2026_52804_MOESM1_ESM.pdf]

Supplementary Figure 1

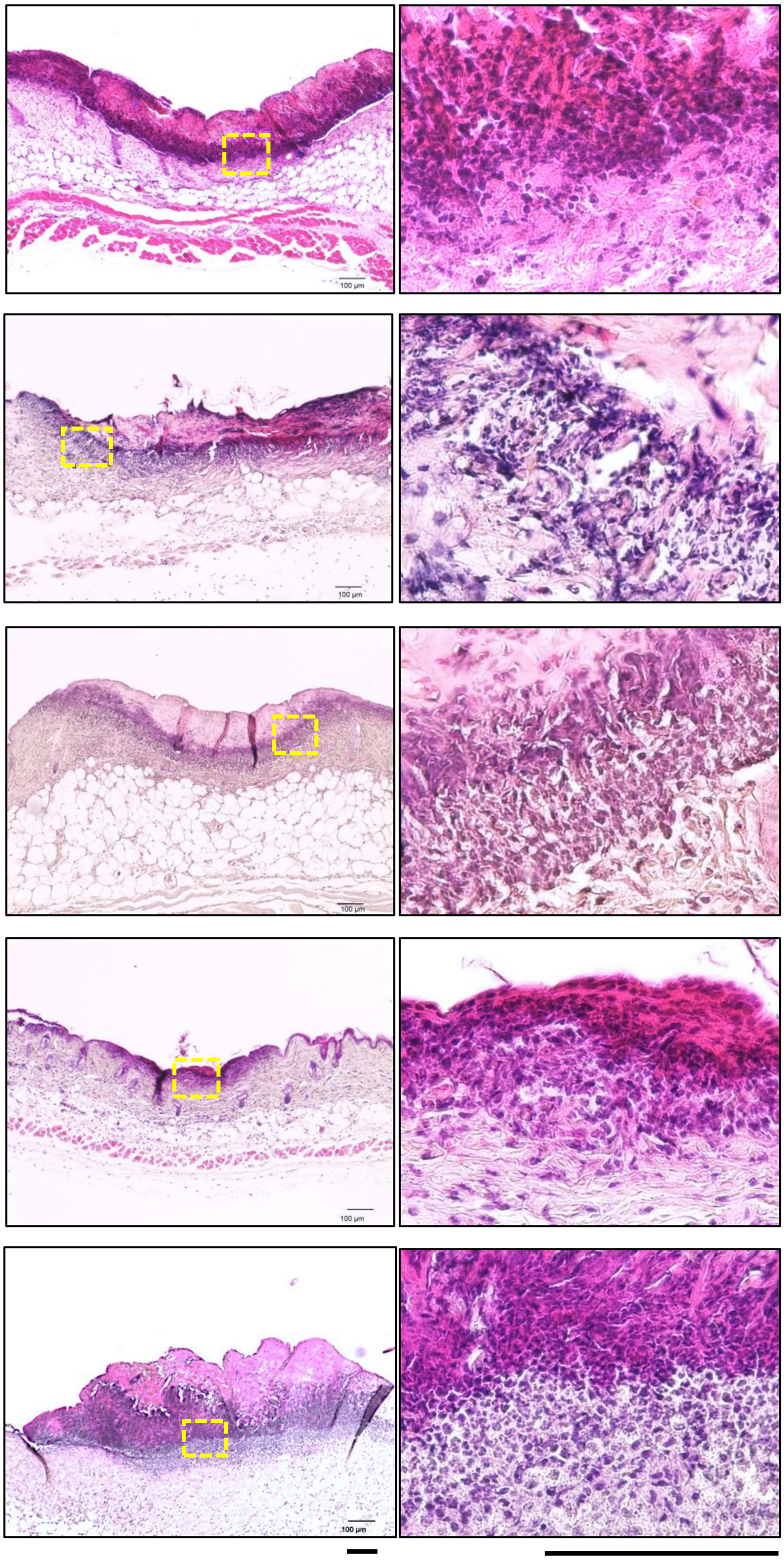

Supplementary Figure 1. Histological images at day 2 from five additional samples (excluding the sample shown in Figure 1).

H&E staining images of dorsal skin wounds at day 2 are shown. Left panels show low-magnification views, and right panels show higher-magnification views of the areas outlined by the yellow dashed boxes in the corresponding left panels. Scale bars, 100 μm.
